# Non-epileptic seizures in autonomic dysfunction as the initial symptom of COVID-19

**DOI:** 10.1007/s00415-020-09904-2

**Published:** 2020-05-26

**Authors:** Kazimierz Logmin, Mohamed Karam, Tanja Schichel, Jens Harmel, Lars Wojtecki

**Affiliations:** 1 Department of Neurology and Neurorehabilitation, Hospital Zum Heiligen Geist, Academic Teaching Hospital of the Heinrich-Heine-University Düsseldorf, von-Broichhausen-Allee 1, 47906 Kempen, Germany; 2 Institute of Clinical Neuroscience and Medical Psychology, Medical Faculty, Heinrich-Heine-University, Düsseldorf, Germany

Dear Sirs,

Originated in Wuhan, severe acute respiratory syndrome coronavirus 2 (SARS-CoV-2) is spreading globally and the number of patients with coronavirus disease 2019 (COVID-19) keeps increasing. The clinical features of COVID-19 include high fever and respiratory syndromes; however, neurological symptoms have also been reported recently [1]. We describe the first case of COVID-19 that initially exhibited non-epileptic seizures (convulsive syncope) in autonomic dysfunction.

On April 2, 2020, a 70-year-old woman was admitted to our hospital due to recurrent non-epileptic seizures/convulsive syncopes. On the day of admission, the patient was found unconscious in her bathroom after a fall. Apart from an enuresis, other stigmata for epileptic seizures, such as delayed reorientation or tongue biting, were not reported. Creatine kinase was elevated in serum, presumably due to the fall. In the last days before admission, the patient had syncopated three times and one of the three syncopes co-occurred with convulsions.

The patient had suffered from syncopes—mainly triggered by sudden pain—over the last years, but not recently. She regularly received methotrexate and etanercept due to psoriatic arthritis. Additional preexistent conditions included neuropathic pain, treated with pregabalin, and paroxysmal atrial fibrillation.

Upon admission, angina pectoris and vertigo were denied. Also fever, cough, a sore throat and loss of gustation or olfaction were not reported. Vital signs did not show relevant abnormalities; body temperature was 36.1 °C, oxygen saturation was 98% on room air, blood pressure was 121/87 mmHg, heart rate was 64 beats per min and breathing rate was 16 breaths per min. Neurological clinical examination and clinical examination of the heart and the lungs did not show relevant (i.e. minor somatosensory) abnormalities. An ECG recorded a sinus rhythm without ischemia-suspicious alterations. Initial laboratory results showed normal leucocytes, but an elevated CRP level with 26.9 mg/L (standard value < 5.0 mg/L). Furthermore, a lymphocytopenia with 1.18/nL (normal 1.26–3.35/nL) should be highlighted. Brain MRI did not show acute alterations, especially no diffusion abnormalities. Three FLAIR hyperintensities were seen as signs of minimal prior ischemic events. In the evening of April 2, 2020, the patient exhibited signs of dyspnea with a desaturation of 82% on room air, an increased breathing rate with 22 breaths per minute and a subfebrile temperature of 37.5 °C. Oropharyngeal swabs were taken, which were negative for the seasonal influenza virus, but positive for SARS-CoV-2. The patient was immediately transferred to the isolation ward on the intermediate care unit and received supportive inhalations (nasal administration of 2 L oxygen). Her immunosuppressive therapy with methotrexate and etanercept was stopped. CSF analysis did not indicate pleocytosis, protein levels were normal, signs of intrathecal immunoglobulin synthesis were absent and oligoclonal bands were negative. SARS-CoV-2-RNA could not be found in the CSF using an RT-PCR assay. Initially, levels of d-dimer-protein and LDH were normal, but both increased in the following days. Procalcitonin was negative throughout the whole hospitalization. The arterial blood gas analysis under nasal administration of 2 L oxygen did not show a decrease of partial oxygen concentration in the blood (ranging from lowest pO_2_ with 77 mmHg to highest pO_2_ with 132 mmHg). Due to the rather stable condition of the patient, antiviral drugs were not administered. Her clinical condition improved progressively, fewer episodes of dyspnea were reported and fever was absent. From day 17 onward, two oropharyngeal swab tests for SARS-CoV-2 were negative on two consecutive days. We complemented the diagnostic procedures;

EEG and Schellong test were normal. Transthoracic echocardiography, long-term ECG and blood pressure monitoring showed no further abnormalities. Heart rate variability was normal. However, the sympathetic skin response was pathological, being an objective sign for autonomic dysfunction. Neurography of N. tibialis, suralis and ulnaris was normal, besides slight prolongation of the F-wave of the tibial nerve. Further convulsive syncopes during the hospitalization were not recorded and on day 21, the patient was discharged from the hospital.

Neurological symptoms in patients with COVID-19 have already been described. In a case series of 214 patients with COVID-19, neurological symptoms were detected in one-third of patients. Symptoms included acute cerebrovascular events, impaired consciousness and muscle injury [1], whereas seizures were rather rare [2]. There is a case report of a meningitis/encephalitis associated with confirmed SARS-CoV-2-RNA in the CSF and transient generalized seizures [3]. To our knowledge, we described the first case of SARS-CoV-2 infection associated with a non-epileptic seizure due to autonomic dysfunction as an initial symptom. Our patient has suffered from convulsive syncopes earlier, but by the time of admission an aggravation of these symptoms was reported. Our patient might be predisposed for convulsive syncopes, since autonomic dysfunction often occurs in patients with psoriatic arthritis [4]. Due to the temporal association with the infection, we think that SARS-CoV-2 infection might be a trigger for the aggravation of the autonomic dysfunction in patients with a certain predisposition. Because of the immunosuppressive therapy with methotrexate and etanercept, our patient was at higher risk for a severe form of COVID-2019, but she recovered well without requiring intensive care. This case shows the importance of considering potential neurological symptoms of a SARS-CoV-2 infection, even if they are atypical or initially unknown.

## Data Availability

Data is available on request.
